# Artificial Intelligence in Cardiovascular Atherosclerosis Imaging

**DOI:** 10.3390/jpm12030420

**Published:** 2022-03-08

**Authors:** Jia Zhang, Ruijuan Han, Guo Shao, Bin Lv, Kai Sun

**Affiliations:** 1 Hohhot Health Committee, Hohhot 010000, China; zhangjia4717@163.com; 2 The People’s Hospital of Longgang District, Shenzhen 518172, China; ruijuanhan@163.com; 3 The Third People’s Hospital of Longgang District, Shenzhen 518100, China; shao.guo.china@gmail.com; 4 Fuwai Hospital, National Center for Cardiovascular Diseases, Beijing 100037, China; blu@vip.sina.com

**Keywords:** artificial intelligence, atherosclerosis, plaque characterization

## Abstract

At present, artificial intelligence (AI) has already been applied in cardiovascular imaging (e.g., image segmentation, automated measurements, and eventually, automated diagnosis) and it has been propelled to the forefront of cardiovascular medical imaging research. In this review, we presented the current status of artificial intelligence applied to image analysis of coronary atherosclerotic plaques, covering multiple areas from plaque component analysis (e.g., identification of plaque properties, identification of vulnerable plaque, detection of myocardial function, and risk prediction) to risk prediction. Additionally, we discuss the current evidence, strengths, limitations, and future directions for AI in cardiac imaging of atherosclerotic plaques, as well as lessons that can be learned from other areas. The continuous development of computer science and technology may further promote the development of this field.

## 1. Introduction

Although modern medical care has increasingly advanced, cardiovascular disease (CVDs) that has an increasing incidence worldwide still poses a serious threat to the quality of human life and health. According to the latest report, CVDs remains the main cause of premature death in most countries, especially low- and middle-income countries [1], which suggests that treatment and prevention of CVDs still need to be improved [2]. Coronary atherosclerosis underlies CAD and major adverse cardiac events (MACEs). Detection of these atherosclerotic plaques, identification of components, and assessment of their risk are essential for the management of patients with cardiovascular disease. Over the past two decades, various medical imaging techniques, including the invasive measurements such as optical coherence tomography (OCT), intravascular ultrasound (IVUS), and noninvasive measurements, such as computed tomography (CT), magnetic resonance imaging (MRI), and ultrasonography (US) have been developed for the assessment of coronary atherosclerosis [3].

With the continuous development of imaging technology and the popularization of imaging examination, massive image datasets have been generated. Meanwhile, big data are a major driver in the development of precision medicine clinicians and researchers alike have more opportunities than ever before to engage in the development and evaluation of novel image analysis algorithms, with the ultimate goal of creating new tools to optimize patient care [4,5]. Artificial intelligence (AI) is regarded as an exciting research topic in multifarious fields, as major advances in AI have occurred in recent years [6]. The application of artificial intelligence to the medical imaging field allows the identification of the information that improve clinical work efficiency. Additionally, AI has recently been propelled to the forefront of cardiovascular medical imaging research [7,8].

The aim of this paper was to focus on research that applied AI for coronary atherosclerotic plaques so as to summarize imaging methods (e.g., OCT, IVUS, CT) and different fields of coronary atherosclerotic plaques (e.g., identification of plaque properties, identification of vulnerable plaque, detection of myocardial function and risk prediction). Finally, we pointed out some current existing problems and future directions.

## 2. Application of AI in Coronary Atherosclerotic Plaque

### 2.1. Overview of Artificial Intelligence

Previous articles have described in detail AI algorithms for cardiovascular imaging [5,9,10,11]. To facilitate understanding of this review, this section provides a short introduction to some terminology. The concept of AI, which instructed machines to have intelligence similar to humans through learning so as to perform specific intelligent tasks [12], discover patterns, and make decisions based on data, was born in the 1950s. Machine learning (ML) is a branch of AI, in which machines or algorithms extract information independently from big data to make predictions without explicit programming [13]. The predictive pattern of ML is similar to traditional regression statistical methods. Still, ML makes predictions based on information obtained from a broad range of big data rather than a limited set of risk factors. Deep learning (DL) is the most advanced branch of ML that most commonly uses a multilayer artificial neural network and a multilayer machine learning model. The distribution characteristics of data are extracted by combining the low-level local image features and converting them to high-level features, and thus developing a model simulating the human brain through a neural network. Nowadays, DL is being used more and more for dealing with large and complex datasets [14]. ML and DL can be classified into two varieties according to whether the labels are clear or not; these two varieties are namely supervised and unsupervised learning. When the label of input data is clear, the supervised learning mode can be selected. When the label of input data is not clear or is lost, the unsupervised mode can be selected to capture and classify the data automatically [15].

### 2.2. Coronary Atherosclerotic Plaque

Coronary atherosclerosis is a common physiological disorder characterized by the formation of fatty streaks proliferation of intimal smooth muscle cells, which eventually leads to coronary artery stenosis [16]. Atherosclerosis (AS) is a complex process that involves interactions between monocyte-derived macrophages, endothelial cells, lymphocyte, and smooth muscle cells [17,18]. The vast majority of CVDs and MACEs usually occurs following the buildup of plaque inside the coronary arteries that supply oxygen-rich blood to the heart muscle. Atherosclerotic lesions start with adaptive thickening of intima characterized by aggregation of intimal smooth muscle cells, which can gradually develop into pathological intima thickening, and are characterized by the presence of cell-free lipid pools. The presence of a necrotic core is the characteristic manifestation of the fibrous aneurysm, where fibrocalcific plaque tends to form following the further development of necrotic core [19]. With complex pathological environmental components, there are notable differences among different evolution stages and compositions of coronary atherosclerotic plaques with regard to outcome [20]. Detection of these atherosclerotic plaques, identification of components, and assessment of their risk are essential for the management of patients with cardiovascular disease.

### 2.3. Characterization of Coronary Atherosclerotic Plaques

Different components of coronary atherosclerotic plaques correspond to different mechanisms, leading to different outcomes [21]. Therefore, accurate identification of plaque components is essential for follow-up treatment. Several previous studies have automatically identified plaque components. 

In the field of noninvasive examinations, Zreik [22] and Rajendra [23] have developed training models to identify plaque calcification in the CCTA automatically. The former uses a multi-task recurrent convolutional neural network (RCNN) to develop an ML model to characterize atherosclerotic plaque properties and coronary stenosis automatically. CCTA images of 81, 17, and 65 patients were used for model training, validation, and testing. The accuracy of this model for plaque characterization (calcification, no calcification, mixing, no plaque) was 0.77 [22]. The latter achieved higher accuracy. He compared the efficacy of different ML algorithms and probabilistic neural networks (PNN), obtaining the best accuracy of 0.89 [23].

Masuda and colleagues proposed a model combining ML with a histogram to detect the characteristics of coronary atherosclerotic plaques (fibrous plaques, fatty plaques) in CCTA. The model shows a significantly higher area under the curve than the traditional method (area under curve 0.92 and 95%, confidence interval 0.86–0.92 vs. 0.83 and 0.75–0.92, *p* = 0.001) [24]. Yamak et al. trained a supervised model using organic phantom plaques fabricated from low-density polyethylene (LDPE) and high-density polyethylene (HDPE). Plaque images from a dual-energy CT scan were used as training data, and the model has shown the ability to identify lipid and calcified plaque by validation analysis in coronary scan images of three patients [25]. 

In the field of invasive examinations, Kim [26] and Sheet [27] attempted to identify plaque components in IVUS images automatically. Kim extracted six image texture features from IVUS images, after which a three-level network classification model was used to classify the coronary plaque into fibrous tissue (FT), fibro-fatty tissue (FFT), necrotic cor (NC), and dense calcium (DC) based on the image texture. The method achieved relatively high sensitivity (82.0%) and specificity (87.1%) in distinguishing between FT/FFT and NC/DC groups [26]. Sheet et al. developed a novel machine-learning-based technique called Stochastic Driven Histology (SDH), which can automatically characterize image components in IVUS images. Validation analysis revealed that SDH is highly consistent with traditional histology in characterizing calcification, fibrotic tissues, and lipids, with 99%, 97%, and 99% accuracy, respectively [27].

There were many studies directed at OCT. Shalev [28] and Xu [29] used a support vector machine (SVM) to identify plaque components in OCT. Shalev trained and validated the model using frozen microscopic data, and the accuracy of calcified plaque recognition achieved 0.97. Xu used a linear SVM classifier to detect unhealthy objects. On this basis, Zhou [30] used more data and improved models to identify lipid plaques and mixed plaques, reaching an accuracy of 91.5% and 78.1%, respectively. Kolluru’s [31] model also trained on frozen images to classify plaques in OCT into four categories, fiber, lipid, calcium, and others. OCT images were paired with frozen images to extract features, after which five-fold cross-validation was performed on the training dataset to optimize classifier parameters. The model achieved an accuracy value that exceeded 90% in all categories. Rico-Jimenez [32] proposed an A-line modeling method to characterize plaques in OCT, which can automatically identify fibrotic plaques and lipid-containing plaques with 85% accuracy. Wilson et al. [33] developed a of convolutional neural network (CNN) in identifying plaque properties in OCT images using line-based modeling methods, learning that CNN can significantly outperform in this task. After that, they proposed a method based on the SegNet deep learning network, proving that the performance of the model was significantly improved compared with the previous method [34]. Athanasiou [35] and Ughi [36] used random forest (RF) classifier to classify atherosclerotic plaques (calcium, lipid pools, fibrous tissue, and mixed plaques) with an accuracy of 80.41% and 81.5%, respectively.

### 2.4. Detection of Coronary Atherosclerotic Plaque

After years of research, a variety of medical imaging techniques have now been used to analyze atherosclerotic plaques. These techniques can detect anatomical and functional abnormalities caused by atherosclerosis, provide detailed information about plaque composition, and even evaluate the risk of atherosclerotic plaques. These methods provide a reference for measuring the severity of coronary atherosclerosis in daily clinical practice and cardiovascular research and have an important role in the diagnosis and treatment of related patients [37]. 

Mainstream noninvasive measurements of coronary atherosclerotic plaques include CT and MRI. The CT can be used to characterize luminal stenosis, assess the component load of plaques and vascular remodeling. As a noninvasive test, it can detect asymptomatic patients with high-risk plaques and stratify the risk of cardiovascular disease [34]. At the same time, CCTA detection of high-risk plaque can identify high-risk patients with MACE events and can be used as an independent predictor of the acute coronary syndrome (ACS) [38]. However, due to the constraints of spatial resolution and radiation dose, CT cannot identify subtle lesions [39]. MRI provides good contrast resolution of soft tissues. In addition to showing the vascular cavity and vascular wall structure, it can also clearly show the plaque load and the progress of plaque bleeding. However, its low spatial resolution and long imaging time make it unsuitable for the diagnosis of active vascular such as coronary arteries. Additionally, there are contraindications in the examination of patients with pacemakers or metals [40], so it is less used for clinical diagnosis of coronary plaque. 

Mainstream invasive measurements of coronary atherosclerotic plaques include OCT and IVUS, which are intravascular techniques that provide a cross-sectional view of the coronary artery. IVUS has special advantages in detecting vulnerable plaques as it can clearly distinguish the properties and composition of different plaques [41]. Yet, IVUS is invasive and expensive, so it is not suitable for a wide population-based screening. OCT provides a greater resolution than IVUS, which clearly shows thin fiber caps; however, some large lipid cores and extravascular elastic layers cannot be observed due to weak tissue penetration [42].

Existing imaging tools can analyze coronary atherosclerotic plaques based on their morphology and structure, but modern precision medicine requires a more detailed analysis of plaque. A large amount of data in the image is inevitably overlooked due to the limitations of the naked eye. Additionally, the methods mentioned above produce large amounts of image data. Working long hours increases the possibility of missed diagnosis or misdiagnosis risks made by radiologist due to the subtle variations in the image that can be easily ignored. Therefore, new imaging diagnosis approaches are urgently required to improve diagnosis efficiency and accuracy by using existing medical imaging data with the ultimate goal of Precision Medicine. 

## 3. Application of AI in Coronary Atherosclerotic Plaque Analysis

### 3.1. Identification of Vulnerable Plaques

Vulnerable plaque rupture is the most common cause of acute coronary syndrome (ACS), which is the most dangerous type of CAD [43]. Pathological features of most vulnerable plaques are characterized by a large necrotic core covered with a thin fibrous cap, as well as abundant inflammatory cells and small amounts of smooth muscle cells [44]. The identification of vulnerable plaques is important for predicting acute cardiovascular events [45]. 

Numerous studies have focused on the field of CCTA. Kolossvary et al. extracted 4400 radiological features from CCTA images of 60 patients by using radiomics and found that 916 features (20.6%) were associated with napkin-ring sign (NRS), of which 440 (9.9%) multiple radiographic features (short-run low-gray-level emphasis, long-run low-gray-level emphasis, the surface ratio of component 2 to the total surface) were more sensitive to high-risk plaques than plaque volume and other conventional quantitative parameters [36]. Then, they performed coronary CT angiography on 21 vitro coronary arteries in the hearts of 7 male donors (average age, 52.3 ± 5.3). Training radiomics-based ML models were used for the diagnosis of advanced atherosclerotic lesions on 333 cross-sections of 95 plaques and evaluation of an additional 112 cross-sections. The results showed that the model was superior to several traditional methods (plaque attenuation pattern scheme in CT angiography cross-sections, histogram-based measurements area of low attenuation (<30 HU), average Hounsfield units of the plaque cross sections) [46]. Recently, they conducted research on 44 plaques of 25 patients. CTA, OCT, IVUS, and NaF 18-PET examinations were performed in all patients. The study found that radiomics outperformed traditional CTA parameters in detecting IVUS low-attenuating plaques, OCT validated thin-cap fibroatheroma (TVFA), and naf18-pet positive lesions (AUC: 0.59 vs. 0.72, 0.66 vs. 0.80, 0.65 vs. 0.87) [43]. They conducted a series of studies, which confirmed the feasibility of using radiomics to detect vulnerable plaques in CCTA, but with similar problems: the studies were based on a single center, using the same scanning and reconstruction parameters, with the small sample size, which may limit the extensive use.

Madani formulated the training model to predict the maximum von Mises stress, which could indicate the risk of plaque rupture, and provide new ideas for the detection of high-risk plaques in the clinical field [44].

Bae [47] and Jun [48] used ML to predict OCT-TCFA in IVUS and compare the accuracy of several different algorithms (SVM, ANN, RF, CNN, etc.). The overall prediction accuracy of the OCT-TFCA exceeds 80%. Sheet et al. collected 13 isolated hearts, using a machine learning framework to identify real necrotic areas of plaques in the IVUS, which is a marker of vulnerable plaques. The speckled appearance of these regions is similar to that of real shaded or severe signal loss regions. Compared with a traditional method such as histological, the sensitivity and specificity of the method were 96.15% and 77.78% [26].

Concerning OCT, Wang et al. [49] proposed a computer-aided method for quantification of fibrous cap (FC) thickness to indicate vulnerable plaques. Liu [50] proposed an automatic detection system of vulnerable plaque for IVOCT images based on a deep convolutional neural network (DCNN). The system is mainly composed of four modules: pre-processing, deep convolutional neural networks (DCNNs), post-processing, and ensemble. The method was intensively evaluated in 300 IVOCT images. The accuracy of the system reached 88.84%, which was a great improvement compared with the previous detection methods.

Fractional flow reserve (FFR) derived from coronary CTA(CT-FFR) is a promising noninvasive maker of coronary physiology and identification of high-risk plaques. Lee, J.M. [51] investigated the utility of noninvasive hemodynamic assessment in the identification of high-risk plaques that caused subsequent acute coronary syndrome (ACS). In this study, the process of deep learning-based CT-FFR is as follows: (1) coronary models, including all epicardial coronary arteries, were constructed by the extraction of vessel centerlines, identification of coronary plaques, and segmentation of lumen boundary along the coronary trees. (2) The flow and pressure in the coronary model were computed by solving the Navier–Stokes equations, using computational fluid dynamics (CFD) methods with assumptions of a rigid wall and a Newtonian fluid [52]. (3) Boundary conditions for hyperemia were derived from myocardial mass, vessel sizes at each outlet, and the response of the microcirculation to adenosine. (4) Combine physiological parameters and fluid mechanics principles with anatomical models to calculate the blood flow and blood pressure of the coronary arteries in the state of maximum hyperemia, and then computed the CT-FFR, change in CT-FFR across the lesion (∆CT-FFR), wall shear stress (WSS) [53]. Additionally, axial plaque stress [54]. The results showed lower CT-FFR and higher ∆CT-FFR, WSS, and axial plaque stress in culprit lesions compared with non-culprit lesions (all *p* values < 0.01), indicating noninvasive hemodynamic assessment enhanced the identification of high-risk plaques that subsequently caused ACS. This study suggests that the integration of noninvasive hemodynamic assessment would enhance the prediction ability for ACS risk and may help provide optimal treatment for those high-risk patients.

Since the recent machine learning algorithm with pixel-level coarse coronary segmentation was insufficient for surface model reconstruction, a new CT-FFR technique with a “Coarse-to-Fine Subpixel” algorithm for lumen contour was proposed to achieve more precise reconstructions. This technique computed subpixel level lumen contour generating the artery centerline after the first coarse coronary segmentation on a pixel level. The new technology would lead to more precise lumen boundary and vessel reconstructions and provide a high diagnostic performance in identifying hemodynamically significant stenosis, “gray zone” lesions, high-risk plaques, and severely calcified lesions.

### 3.2. Assessment of Myocardium

At present, the gold standard for the diagnosis of myocardial specific ischemia is the fractional flow reserve (FFR), which can guide interventional therapy and improve the prognosis of patients with CAD [55]. The study showed that the characteristics of coronary plaque can also characterize myocardial ischemia [56]. Dey et al. [57] combined quantitative stenosis, plaque burden, and myocardial quality into a comprehensive risk score to predict the impairment of MFR through enhanced integrated machine learning algorithms. The experiment demonstrated that arterial non-calcified plaque (NCP) load and the approach combined CTA quantitative stenosis and the above comprehensive score significantly improved the identification of vascular dysfunction in the downstream compared with stenosis. Next, they explored the possibility of effectively combining CTA clinical data, quantitative stenosis, and plaque indicators with AI to predict specific ischemia. A total of 254 patients were enrolled, and quantitative plaque analysis was used to predict lesion-specific ischemia, with a final AUC of 0.84 [58]. Other experts tried to combine AI-based plaque analysis tools with CT-FFR to improve the prediction of myocardial ischemia. Teams of Gaur [59], von Knebel Doeberitz [60], and Kawasaki [61] used FFR as the gold standard and proposed machine-learning-based approaches combining CCTA plaque analysis and CT-FFR. Their results showed that the predictive ability of local ischemia was 0.90, 0.93 and 0.835, respectively, which was superior to that of traditional CCTA narrow grading.

### 3.3. Risk Prediction

The risk assessment of cardiovascular disease depends on a variety of factors, such as sex, age, weight, smoking, drinking, and so on [62]. Moreover, the risk level of patients with diabetes [63], elevated cholesterol, or blood pressure [64] also tend to differ. Different morphologies of plaques in medical imaging are significant for cardiovascular risk stratification [65,66]. Therefore, another important application of AI algorithms in the medical field is the prediction of cardiovascular disease risk.

In the field of IVUS, Araki presented a model to assess the risk of coronary heart disease by combining the IVUS grayscale plaque morphology and carotid B-mode ultrasound carotid intima-media thickness (cIMT) based on SMV, which is a marker of subclinical atherosclerosis [67]. The team then added plaque major component analysis to the model, proposed an SVM framework based on plaque morphology and major component (PAC) to assess coronary plaque risk, AUC = 0.98 [64]. The same team established an ML model by merging the plaques texture-based with the wall-based measurement features (coronary calcium area, coronary vessel area, coronary lumen area, coronary atheroma area, coronary wall thickness, and coronary wall thickness variability), which improved the accuracy of risk assessment by about 6% compared with the plaques texture-based information [68]. Cao [69] proposed a neural network-based method to determine the critical point of a vulnerability index, which distinguishes the fragile plaque from the stable plaque, AUC = 0.7143. Zhang [70] reported a machine learning approach for predicting the location and type of high-risk coronary plaque in patients treated with statins therapy.

Considering the studies of risk predicting focuses on CCTA in the field of noninvasive examination, van Assen [71] used ML to automatically extract plaque information so as to predict MACEs, AUC = 0.924. Van Rosendael [72] trained the ML model using coronary artery stenosis and plaque component information to predict mortality in patients with CAD, AUC = 0.771, beyond other conventional risk scores. Johnson [73] evaluated the prognosis of 6892 CCTA patients by ML, reporting that the AUC for all-cause death, CAD death, coronary heart disease death, and nonfatal myocardial infarction was 0.77, 0.72, 0.85, and 0.79, respectively. Motwani et al. [74] further added clinical risk factors to predict 5-year all-cause mortality in patients with CAD. They evaluated 25 clinical and 44 CCTA parameters, and ML showed higher AUC than other models (segment stenosis score, segment involvement score, modified Duke Index, Framingham risk score). Han [75] and Kigka [76] used ML to predict the rapid development of coronary plaque, which was thought to be associated with cardiovascular events [77,78], revealing the prediction accuracy of 0.81 and 0.84, respectively. Table 1 displays the application of AI in coronary atherosclerotic plaque analysis.

## 4. Limitations

There are still limitations in this field. In the research of ML used in coronary atherosclerotic plaques analysis, more prominent problems are the following two points: first, in almost all studies, data derived from a single research center or an old public dataset make it difficult to cover patients with different conditions and scanning parameters, making the training model difficult to satisfy the complex scenarios of clinical. Larger, rich public datasets should be established in the future for higher-quality research. Second, most of the research in this field takes the diagnostic opinion of artificial experts as the standard, lacking validation of the gold standard of pathology; therefore, the individual bias of experts may affect the accuracy of the final model. Future research on artificial intelligence for coronary atherosclerotic plaque analysis should be based on more big data; additionally, multicenter research is necessary to provide better algorithmic models.

## 5. Conclusions

**Figure 1:**
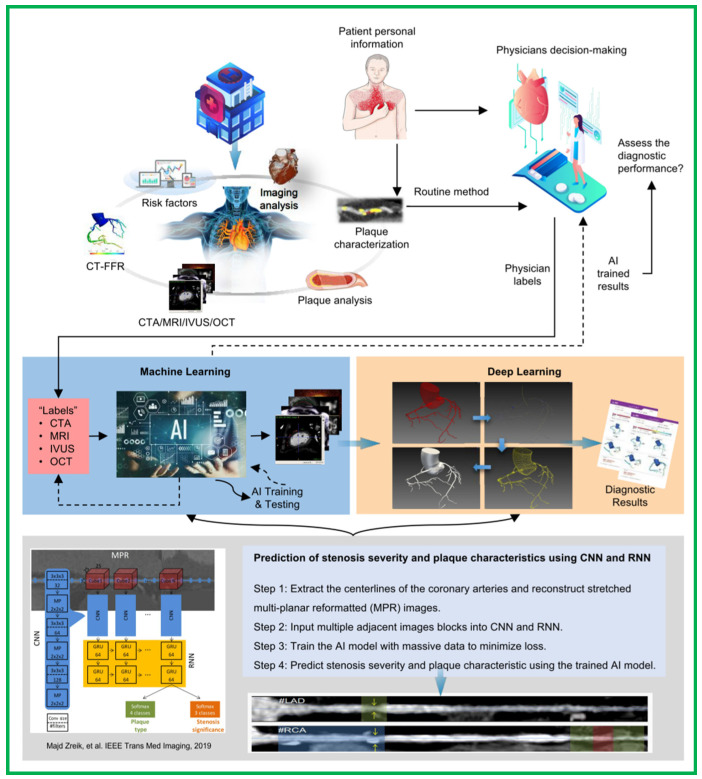
AI in cardiovascular atherosclerosis imaging. A proposed workflow for the incorporation of machine learning and deep learning analysis of imaging modalities in clinical practice. AI analysis can reduce the analysis time and provide automated recommendations to physicians regarding diagnosis and downstream treatment decision making. The workflow brings in a promising algorithm, based on a recurrent convolutional neural network, for the automatic detection and characterization of coronary artery plaque, as well as the detection and characterization of the anatomical significance of coronary artery stenosis.

In summary, artificial intelligence has the potential to expand and improve medical technologies for better patient care, by reducing the analysis time and provide automated recommendations to physicians regarding diagnosis and downstream treatment decision making. A proposed workflow for the incorporation of machine learning and deep learning analysis of imaging modalities in clinical practice. The workflow brings in a promising algorithm, based on a recurrent convolutional neural network, for automatic detection and characterization of coronary artery plaque, as well as detection and characterization of the anatomical significance of coronary artery stenosis. The areas of AI-based cardiovascular imaging covered range from imaging analysis (e.g., image segmentation, automated measurements, and eventually, automated diagnosis) to diagnostic imaging, including identifying plaques, assessing plaque vulnerability, myocardial hemodynamic evaluation, such as deep learning-based CT-FFR, and carrying out risk prognosis assessments. Specifically, the ability of the AI algorithms to make more accurate diagnoses is useful for physicians to detect diseases earlier in their course to plan for the right treatment action (Figure 1). With the development of computer technology, bioengineering, and medical imaging technology, the future of AI in cardiovascular imaging is bright as the collaboration between investigators and clinicians will have great benefits.

## Figures and Tables

**Table 1:** Application of AI in coronary atherosclerotic plaque analysis.

Authors	Vascular Segments	Year	The Method Applied	Outcomes	Advantages	Disadvantages
Athanasiou	Plaques	2011	OCT	Random forest (RF), accuracy of 80.41%	Random forest (RF) classifier to classify atherosclerotic plaques (calcium, lipid pools, fibrous tissue, and mixed plaques)	Invasive
Wang	Vulnerable plaques	2012	Fibrous cap (FC)	Proposed a computer-aided method for quantification of fibrous cap (FC) thickness to indicate vulnerable plaques	A method for quantification of fibrous cap (FC) thickness	Invasive
Sheet D	Coronary plaque	2013	IVUS	Validation analysis revealed that SDH is highly consistent with traditional histology in characterizing calcification, fibrotic tissues, and lipids, with 99%, 97%, 99% accuracy, respectively	Developed a novel machine-learning-based technique called Stochastic Driven Histology (SDH), which can automatically characterize image components in IVUS images	Invasive, the small number of observation
Ughi	Plaques	2013	OCT	Random forest (RF), accuracy of 81.5%%	Random forest (RF) classifier to classify atherosclerotic plaques (calcium, lipid pools, fibrous tissue, and mixed plaques)	Invasive
Yamak D	Coronary plaque	2014	Non-calcified coronary atherosclerotic plaque. Characterization by Dual Energy Computed Tomography	Learning approaches were explored as a more advanced mathematical analysis to use additional information provided by DECT	Three models (ANN, RF and SVM)	The small number of observations is the other limitation of this study
Xu M	Atherosclerotic heart disease	2014	OCT	A linear SVM classifier to detect unhealthy objects	The system classifies the image from healthy and unhealthy subjects automatically by utilizing texture features	Invasive
Gaur	Coronary	2016	Coronary CTA stenosis, plaque volumes, FFRCT, and FFR were assessed	Redictive ability of local ischemia was 0.90	Coronary atherosclerotic plaque and FFRCT assessment improve the discrimination of ischaemia	Did not confirm plaque findings by intravascular ultrasound
Shalev R	Coronary plaque	2016	OCT	Rained and validated the model using frozen microscopic data, and the accuracy of calcified plaque recognition achieved 0.97	Regions for extraction of sub-images (SI’s) were selected by experts to include calcium, fibrous, or lipid tissues	Invasive
Rico-Jimenez	Aining plaques	2016	OCT	An A-line modeling method to characterize plaques in OCT, which can automatically identify fibrotic plaques and lipid-containing plaques with 85% accuracy	Automatically identify fibrotic plaques and lipid-containing plaques	Invasive
Kolossváry M	Coronary vulnerable plaques	2017	Features are superior to conventional quantitative computed tomographic metrics to identify coronary plaques with napkin-ring sign	Radiomics and found that 916 features (20.6%) were associated with napkin-ring sign (NRS), of which 440 (9.9%) multiple radiographic features (short-run low-gray-level emphasis, long-run low-gray-level emphasis	High-risk plaques, napkin-ring sign	The true prevalence of the NRS is considerably smaller compared with non-NRS plaques in a real population
Kim G	Coronary plaque	2018	Plaque components were classifed into FT, FFT, NC, or DC using an intensity-based multi-level classifcation model	The classifers had classifcation accuracies of 85.1%, 71.9%, and 77.2%, respectively	Three diferent nets. Net 1 diferentiated low-intensity components into FT/FFT and NC/DC groups. Then, net 2 subsequently divided FT/FFT into FT or FFT, NC or DC via net 3	Invasive, it did not acquire signifcant classifcation results compared with VH
Kolluru	Classify plaques in OCT	2018	OCT	The model achieved an accuracy value that exceeded 90% in all categories.	Model also trained on frozen images to classify plaques in OCT into four categories, fiber, lipid, calcium, and others	Invasive
Wilson	Plaques	2018	OCT	Convolutional neural network (CNN) in identifying plaque properties in OCT images using line-based modeling methods, learning that CNN can significantly outperform in this task	A method based on the SegNet deep learning network	Invasive
Zreik M	Coronary artery plaque	2019	A recurrent CNN for automatic detection and classification of coronary crtery plaque and stenosis in coronary CT angiography	For detection and characterization of coronary plaque, the method was achieved an accuracy of 0.77	Three-dimensional convolutional neural network and neural networkautomatic detection and classification of coronary artery plaque and stenosis are feasible	Coronary artery bifurcations were not manually annotated and the network was not trained to detect these as a separate class
Rajendra	Coronary artery plaque	2019	Seven features are extracted from the Gabor coefficients: energy, and Kapur, Max, Rényi, Shannon, Vajda, and Yager entropies	The features acquired were also ranked according to F-value and input to several classifiers, an accuracy, positive predictive value, sensitivity, and specificity of 89.09%, 91.70%, 91.83% and 83.70% were obtained	Automated plaque classification using computed tomography angiography and Gabor transformationscan be helpful in the automated classification of plaques present in CTA images	The database was limited to only 73 patients. Furthermore, no quantitative calcium score was calculated
Masuda T	Coronary artery plaque	2019	Recorded the coronary CT number and 7 histogram parameters (minimum and mean value, standard deviation (SD), maximum value, skewness, kurtosis, and entropy) of the plaque CT number	Coronary CT number (0.19) followed by the minimum value (0.17), kurtosis (0.17), entropy (0.14), skewness (0.11), the mean value (0.11), the standard deviation (0.06), and the maximum value (0.05), and energy (0.00)	The machine learning was superior the conventional cut-off method for coronary plaque characterization using the plaque CT number on CCTA images	A small single-protocol study and only the performance of the machine learning algorithm was evaluated
Kolossváry M.	Coronary vulnerable plaques	2019	Diagnosis of advanced atherosclerotic lesions on 333 cross-sections of 95 plaques and evaluation of an additional 112 cross-sections	The results showed that the model was superior to several traditional methods.	Radiomics-based ML models outperformed expert visual assessment and histogram-based methods in the identification of advanced atheroscle radiomics-based machine learning rotic lesion	Limited spatial resolution of coronary CT angiography
Kolossváry M.	Coronaryvuinerable plaques	2019	Radiomics outperformed traditional CTA parameters in detecting IVUS low-attenuating plaques, OCT validated thin-cap fibroatheroma (TVFA) and naf18-pet	CTA, IVUS, OCT, positive lesions (AUC: 0.59 vs. 0.72, 0.66 vs. 0.80, 0.65 vs. 0.87)	Coronary CTA radiomics showed a good diagnostic accuracy to identify IVUS-attenuated plaques and excellent diagnostic accuracy to identify OCT-TCFA	Our results of the general populations are limited, multicenter longitudinal studies are warranted
von Knebel	Coronary	2019	ICA, CT-FFR	Redictive ability of local ischemia was 0.93	CCTA-derived plaque markers and CT-FFR have discriminatory power to differentiate between hemodynamically significant and non-significant coronary lesions	Did not systematically correlate our findings on CCTA with an invasive reference standard
Kawasaki	Coronary	2019	CT-FFR	rRdictive ability of local ischemia was 0.835	CCTA features and functional CT-FFR was helpful for detecting lesion-specific ischemia	Did not evaluate the influence of CT image quality on the CT-FFR measurements
Liu	Vulnerable plaques	2019	IVOCT images based on a deep convolutional neural network (DCNN)	Automatic detection system of vulnerable plaque for IVOCT images based on a deep convolutional neural network (DCNN). The accuracy of the system reached 88.84%	Intravascular optical coherence tomography (IVOCT)	Invasive

## Abbreviations

AI	Artificial intelligence
MACEs	Major adverse cardiac events
CVDs	Cardiovascular disease
CAD	Coronary artery disease
OCT	Optical coherence tomography
CT	Computed tomography
MRI	Magnetic resonance imaging
IVUS	Intravascular ultrasound
AS	Atherosclerosis
ACS	Acute coronary syndrome
ML	Machine learning
DL	Deep learning
ROI	Region of interest
